# Elastic stable intramedullary nail combined with Kirschner wire (E-K) technique for treating pediatric distal tibial diaphyseal metaphyseal junction (DTDMJ) fractures

**DOI:** 10.3389/fped.2024.1333652

**Published:** 2024-04-16

**Authors:** Yunlong Liu, Sheng Ding, Yancai Yang

**Affiliations:** Department of Pediatric Surgery, Ningbo Women and Children's Hospital, Ningbo, China

**Keywords:** Kirschner wire, DTDMJ, tibial fractures in children, elastic stable intramedullary nails, pediatric

## Abstract

**Objective:**

Elastic stable intramedullary nail (ESIN) is a commonly used method for treating diaphyseal fractures of the tibia, but its application in Distal Tibial Diaphyseal Metaphyseal Junction (DTDMJ) fractures has been a subject of controversy. This study aims to evaluate the clinical efficacy of the Elastic stable intramedullary nail-Kirschner wire (E-K) technique in treating pediatric DTDMJ fractures, providing better clinical decision-making for clinicians in diagnosing and treating such fractures.

**Methods:**

We conducted a retrospective analysis of patients aged 3–9 years who received treatment at our hospital from January 2019–January 2021 for distal tibial diaphyseal metaphyseal junction (DTDMJ) fractures. Based on their surgical procedures, they were categorized into the Elastic Stable Intramedullary Nail—Kirschner wire group (E-K) and the ESIN group. Demographic data, surgical duration, clinical outcomes, complications, and imaging data were recorded.

**Results:**

The study included a total of 57 patients, with 24 cases in the E-K group and 33 cases in the ESIN group. There were 30 males and 27 females. The average age was (6.25 ± 1.59) years in the E-K group and (6.27 ± 1.48) years in the ESIN group. There were no significant differences between the two groups in terms of gender, age, weight, time from injury to surgery, follow-up time, side of injury, associated injuries, nail site infection, deep infection, and nail removal time (*P* > 0.05). Neither group experienced nonunion or refracture. The E-K group exhibited significantly lower coronal and sagittal plane angular values at the final follow-up compared to the ESIN group (*P* < 0.001). In the E-K group, the final follow-up coronal plane angle was 2.67 (1.09)°, while in the ESIN group, it was 6.55 (2.05)°. The final follow-up sagittal plane angle was 3.12 (1.54)° in the E-K group and 7.58 (1.48)° in the ESIN group. Both groups showed good alignment in the initial postoperative x-rays, with no statistically significant differences. However, during clinical healing, the ESIN group exhibited significant displacement, whereas the E-K group had minimal displacement, demonstrating a significant statistical difference (*P* < 0.001). There was a statistically significant difference in the AOFAS joint function assessment between the two groups (*P* = 0.027).

**Conclusion:**

The E-K technique is a viable option for treating DTDMJ fractures in pediatric patients, with well-established clinical efficacy. Its advantages include a straightforward surgical procedure, safety, and a low incidence of severe complications.

## 1 Introduction

Tibial fracture is a common type of long bone fracture in children (1–4), accounting for approximately 15% of cases (2). These fractures occur in both male and female children, although the incidence may vary slightly between boys and girls. Around 50%–70% of pediatric tibial fractures occur in the distal one-third of the tibia, while 19%–39% occur in the middle one-third of the tibia (5, 6).

Pediatric distal tibial diaphyseal metaphyseal junction (DTDMJ) fractures represent a specific type of tibial fracture in children, often presenting as oblique, comminuted, or transverse fractures (7, 8). This region's fractures are not typically treated with ESIN and have historically been managed with open reduction and plate fixation, while some open fractures may be treated with external fixation devices. In recent years, the application of Elastic Stable Intramedullary Nails (ESIN) has gradually been introduced. ESIN offers advantages such as minimally invasive surgery, shorter hospitalization, faster healing, and early weight-bearing (8). However, ESIN treatment has also presented a range of issues, including axial instability, loss of reduction, and angular deformities.Open reduction and plate fixation can achieve an ideal anatomical reduction of the fracture but involve significant surgical trauma and impact the blood supply to the fracture site, which can further affect the healing process. External fixation treatment is relatively challenging in terms of care, costly, and associated with a higher rate of complications (9, 10).

In recent years, in order to find a minimally invasive and simple yet effective method for reducing and internal fixation in the treatment of such fractures, we attempted to apply the technique of combining elastic nails with Kirschner wires to fractures at the tibial diaphyseal-metaphyseal junction. Considering the inability of elastic nails to generate interfragmentary compression within the medullary canal at this location, we enhanced the stability by combining percutaneous fixation with Kirschner wires and external immobilization with a cast, resulting in preliminary favorable treatment outcomes.

## 2 Materials and methods

### 2.1 Clinical and radiographs evaluation

The study retrospectively analyzed the patients aged 3–9 with DTDMJ fractures who underwent treatment at our hospital from January 2019–January 2021. Based on the surgical methods, the study population was divided into the E-K group and the ESIN group. Inclusion criteria: (1) Patients diagnosed with DTDMJ fractures; (2) Aged between 3 and 9 years old. Exclusion criteria: (1) Gustilo grade 2 and 3 open fractures were excluded. (2) Patients with pathological fractures, neuromuscular disorders, metabolic diseases, previous tibial fractures, or those who had undergone internal fixation. (3) Patients with a follow-up time of less than 24 months or incomplete medical records. This study obtained informed consent from the parents and received approval from the institutional ethics review committee. DTDMJ is defined based on “The AO Pediatric Comprehensive Classification of Long Bone Fractures” (7, 8).

During the perioperative period, the following assessments were made using anteroposterior and lateral x-ray images of the tibia and fibula: Evaluation of the location and displacement of DTDMJ fracture, as well as the presence of fibular fracture. Fracture line length less than twice the bone width was defined as a mild oblique fracture. Fracture displacement was considered unstable when it exceeds two-thirds of the diameter and/or has an angulation of 30°. Malunion was defined as coronal or sagittal plane angulation greater than 4°, and the limb length discrepancy (LLD) of ≥1 cm was considered as a complication. During follow-up, the activity range of the ankle joint was evaluated using the AOFAS (American Orthopaedic Foot & Ankle Society) criteria, and the AOFAS score and any complications at the last follow-up were recorded. Our clinical healing assessment criterion was x-ray evidence of bridging callus at the fracture site in three directions (11–13).

### 2.2 Surgical treatment

**Figure 1:**
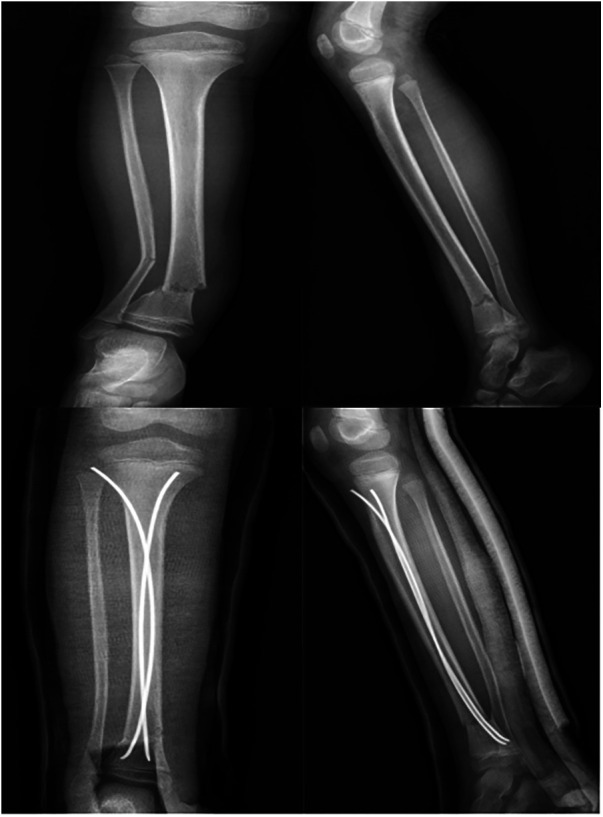
Case1: A 5-year-old boy had a fracture of the distal tibial diaphyseal metaphyseal junction(DTDMJ). X-ray films showed coronal valgus deformity of the distal tibiofibular fracture with displacement. The coronal valgus angulation remained uncorrected after treatment with the ESIN technique.

**Figure 2:**
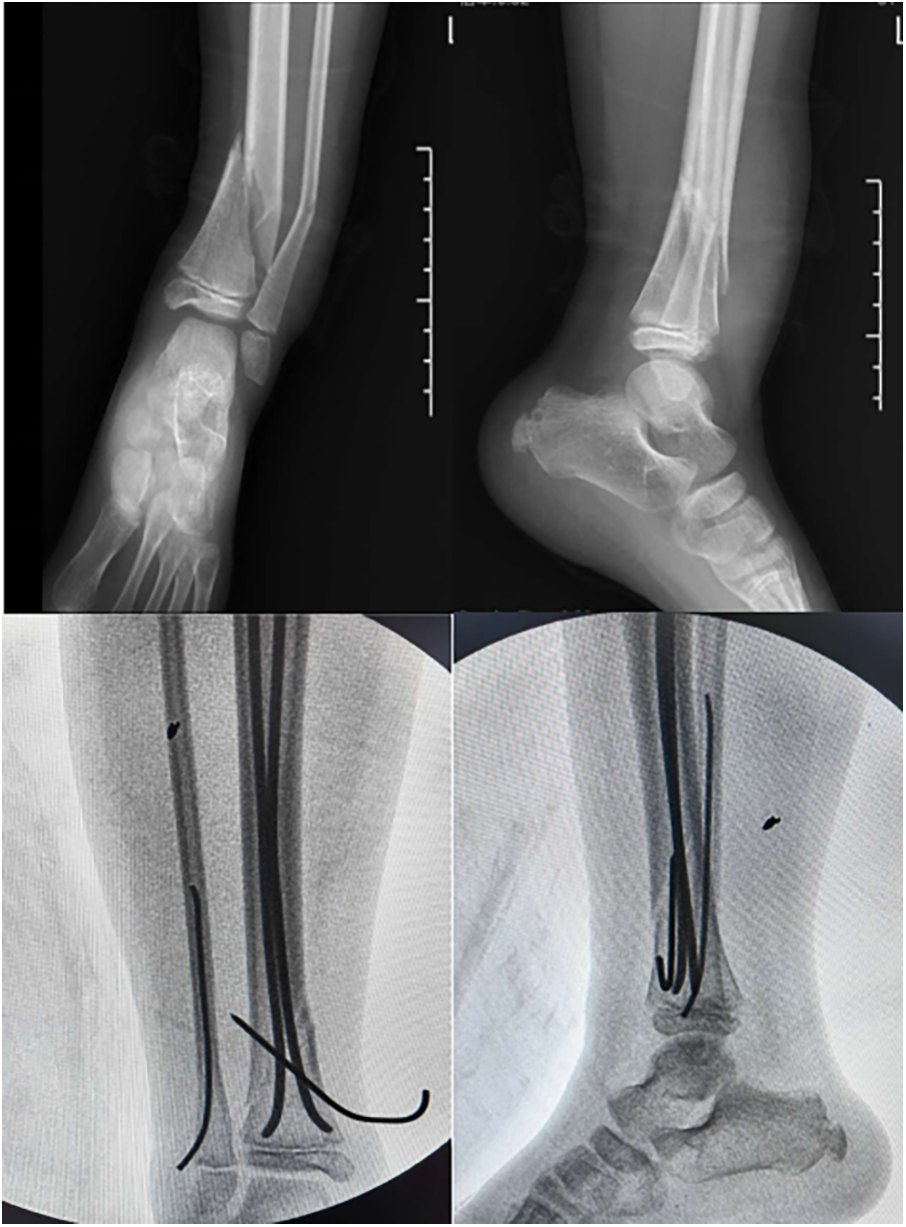
Case2: A 6-year-old girl presented with a fracture of the distal tibial diaphyseal metaphyseal junction(DTDMJ). X-ray films showed coronal varus deformity of the distal tibiofibular fracture with displacement. After treatment with E-K technique, the coronal varus Angle of the distal tibiofibular fracture was completely corrected.

The surgeries were performed by experienced senior Pediatric orthopedic surgeons. The pediatric patients were placed in a supine position under general anesthesia. The choice of ESIN (Elastic Stable Intramedullary Nailing) was based on the narrowest width of the tibial shaft medullary canal, with the diameter of a single intramedullary nail being 30%–40% of the minimum width of the medullary canal. The ESIN was pre-bent into an arc, with the arc height being approximately 3–5 times the minimum diameter of the tibial medullary canal. Two ESINs with the same curvature were inserted, with their apex positioned at the site of the fracture. A small incision was made on the medial or lateral aspect of the proximal tibia, approximately 1–2 cm from the epiphyseal plate. The periosteum was bluntly dissected, the bone membrane was incised, and a bone trocar was inserted at a 45° angle into the medullary canal. Two elastic intramedullary nails were inserted into the medullary canal, reaching the site of the fracture. Repositioning of the fracture ends was achieved using 1–2 Kirschner wires (2.0 mm) for leverage and manipulation. Once satisfactory fracture reduction was confirmed under fluoroscopy, Kirschner wires were percutaneously inserted to secure the proximal and distal fracture ends. The direction of the intramedullary nails was adjusted, and they were advanced. After successful fracture reduction, the intramedullary nails were continued to be advanced, ensuring they extended at least 1 cm beyond the distal epiphyseal plate. The intramedullary nails were then fixed in place, and the nail tails were bent for easy removal of the nails and to prevent migration. The nail tips were cut, leaving approximately 1–2 cm of the nail tails, slightly rotated dorsally.The Kirschner wires were bent externally and partially broken, followed by an x-ray examination to confirm the correct positioning of both the intramedullary nails and Kirschner wires. Once confirmed, the incision was closed. External immobilization with a cast was applied, and after 4–6 weeks, when bridging callus was observed in two or more directions at the fracture site, the Kirschner wires could be removed. The cast continued to be applied until clinical healing of the fracture (Figures 1, 2).

### 2.3 Statistical analysis

We conducted the statistical analysis using SPSS statistical package program (version 19.0, SPSS Inc., Chicago, Illinois, USA). Categorical data were assessed with the Chi-square (*χ*²) test, while continuous data were analyzed using Student's *t*-test. In cases with smaller sample sizes in specific groups, the Fisher exact test was employed. The results are presented as mean ± SD (range), median (range), or *n* (%). Statistical significance was considered at *p* < 0.05.

## 3 Results

As shown in Table 1, the E-K group included 24 patients, comprising 14 males and 10 females, while the ESIN group included 33 patients, with 16 males and 17 females (*P* = 0.641). The average age of patients in the E-K group was 6.25 ± 1.59 years, and in the ESIN group, it was 6.27 ± 1.48 years (*P* = 0.961). Both groups were followed up for at least 2 years (*P* = 0.068). There were no significant differences between the two groups in terms of gender, age, weight, time from injury to surgery, follow-up duration, injured side, and associated injuries (Table 1).

**Table 1:** Patient demographics.

	E-K	ESIN	*P*
	*N* = 24	*N* = 33	
Age (years)	6.25 (1.59)	6.27 (1.48)	0.961
Gender			0.641
Male	14 (58.3%)	16 (48.5%)	
Female	10 (41.7%)	17 (51.5%)	
Weight (kg)	22.8 (4.24)	22.4 (3.09)	0.678
Side			0.359
Right	10 (41.7%)	19 (57.6%)	
Left	14 (58.3%)	14 (42.4%)	
Presence of fibular fracture			1.000
No	1 (4.17%)	1 (3.03%)	
Yes	23 (95.8%)	32 (97.0%)	
Concomitant injuries			1.000
No	23 (95.8%)	32 (97.0%)	
Yes	1 (4.17%)	1 (3.03%)	
From injury to surgery (days)	2.04 (0.36)	2.06 (0.35)	0.843
Operation time (mins)	36.8 (5.27)	36.6 (4.76)	0.892
Radiological union	1.92 (0.24)	1.95 (0.23)	0.552
Follow-up time	26.2 (2.57)	27.4 (2.21)	0.068

E-K: Elastic stable intramedullary nail combined with Kirschner wire.

ESIN: Elastic stable intramedullary nail; Concomitant injuries: head, thoracic and abdominal, pelvic injuries.

As shown in Table 2, both groups did not experience nonunion or refracture. The final follow-up angular values in the coronal and sagittal planes were significantly lower in the E-K group compared to the ESIN group (*P* < 0.001). In the E-K group, the coronal plane angular value at the final follow-up was 2.67 (1.09)°, while in the ESIN group, it was 6.55 (2.05)°. The final follow-up sagittal plane angles were 3.12 (1.54)° in the E-K group and 7.58 (1.48)° in the ESIN group. Both groups exhibited good alignment on initial postoperative x-rays, with no statistically significant differences. However, at the point of clinical healing, the ESIN group showed noticeable displacement compared to the E-K group, with a significant statistical difference (*P* < 0.001). There was a statistically significant difference in the AOFAS joint function assessment between the two groups (*P* = 0.027) (Table 2).

**Table 2:** Radiological and clinical outcomes.

	E-K	ESIN	*P*
	*N* = 24	*N* = 33	
First postoperative radiograph			
Coronal Angulation (degree)	2.58 (0.78)	5.27 (2.04)	<0.001
Sagittal Angulation (degree)	2.83 (0.96)	6.39 (1.69)	<0.001
Fracture position (degree)	1.32 (1.83)	0.92 (0.03)	0.288
Radiological union radiograph			
Coronal Angulation (degree)	2.67 (1.09)	6.55 (2.05)	<0.001
Sagittal Angulation (degree)	3.12 (1.54)	7.58 (1.48)	<0.001
Fracture position (degree)	0.94 (0.03)	0.88 (0.05)	<0.001
AOFAS:			**0** **.** **027**
Excellent	22 (91.7%)	21 (63.6%)	
Good	2 (8.33%)	4 (12.1%)	
Pass	0 (0.00%)	7 (21.2%)	
Poor	0 (0.00%)	1 (3.03%)	
Hardwarae removal (months)	6.88 (0.88)	6.67 (0.96)	0.398
Non-union	24 (100%)	33 (100%)	-
Deep infection	24 (100%)	33 (100%)	-
Refracture	24 (100%)	33 (100%)	-
Pin tract infection	24 (100%)	33 (100%)	-
LLD (mm)	6.62 (1.64)	4.18 (1.63)	<0.001

LLD, limb length discrepancy; AOFAS, American orthopedic foot and ankle society.

There were no significant differences between the two groups in terms of nail tract infection, deep infection, or nail removal time. Limb length discrepancy (LLD) was more pronounced in the ESIN group (4.18 ± 1.63 mm) compared to the E-K group (6.62 ± 1.64 mm) (*P* < 0.001). However, in both groups, LLD was less than 1.0 cm, and no gait abnormalities were observed during the follow-up.

## 4 Discussion

Pediatric tibia fractures are the third most common long bone fractures, following forearm and femur fractures (14). The incidence of tibia fractures in both boys and girls is increasing year by year. The average age is 8 years, and the incidence does not significantly vary with age. 70% of pediatric tibia fractures occur as isolated fractures, with 30% occurring in conjunction with a same-side fibula fracture. Tibia fractures occur in the distal third in 50%–70% of cases and in the middle third in 19%–39% of cases (5–7).

Children's bones are in a stage of growth and development, and they have a strong capacity for bone remodeling and correction after fractures. For the vast majority of cases, conservative treatment is highly effective and is the preferred method for treating pediatric tibia fractures (14). However, for severe displaced fractures caused by high-energy injuries such as falls from heights, traffic accidents, open fractures, comminuted fractures, and cases involving instability and associated injuries, achieving acceptable stability and alignment through conservative treatment can be challenging. Common complications in such cases include limb length discrepancy, angular deformities, and rotational malalignment (15). In recent years, there has been a growing trend in literature reporting cases of pressure sores, compartment syndrome, nursing difficulties, and psychological issues. Non-surgical treatment of long spiral diaphyseal tibia fractures may result in a recurrence rate of 2%–5%. Uludağ and others reported 65 cases of unstable diaphyseal tibia fractures in children treated with closed reduction and cast immobilization, with 25 cases experiencing recurrence, and approximately one-third of these children were under 11 years of age (16). Recurrence often occurred during the second to third week of cast immobilization. Some researchers also suggest that if there is more than a 5° internal rotation at the distal tibia fracture site, secondary reduction may be necessary, as residual angular deformity is a primary cause of nonunion in these fractures (17–19).

The ESIN technique was first reported by Ligier at Nancy Hospital in France in 1983 and has since been widely adopted in North America and Europe (14). Around the year 2000, cases related to ESIN started to be reported in China. This technique, known for its advantages of minimal trauma, a short learning curve, and early weight-bearing for children, gradually found application in the treatment of long bone diaphyseal fractures in pediatric patients. In recent years, there has been a growing trend in the use of ESIN for pediatric tibia diaphyseal fractures, particularly for transverse and short oblique fractures. Researchers such as Ke evaluated the outcomes of ESIN treatment in 16 cases of tibia diaphyseal fractures, all of which achieved pain-free healing within five years, without any complications such as growth inhibition or refracture (20).

Pediatric DTDMJ fractures (7, 8) represent a specific type of tibial fracture in children. These fractures pose challenges for surgical reduction and fixation due to their proximity to the epiphyseal plate, axial instability, local force imbalances, and irregular fracture lines (21, 22). In 2014, Cravino and colleagues (23) first reported the use of ESIN in the treatment of 18 cases of tibial diaphyseal-metaphyseal junction fractures. The results showed that this minimally invasive technique was as effective as in diaphyseal fractures and had fewer complications.

In pediatric DTDMJ fractures, ESIN usually cannot provide effective fixation through its elastic modulus and cross-stress. Postoperatively, complications such as fracture redisplacement, non-union, angular deformities, and shortening displacement are more likely to occur (20). In our clinical study, we found that children in the ESIN group showed noticeable coronal and sagittal angular deformities and worsened alignment on initial postoperative x-rays and during clinical healing of the fracture compared to the E-K group, with statistically significant differences. Therefore, we believe that, in combination with the rigid characteristics of Kirschner wires, leveraging Kirschner wires for fracture reduction and percutaneous fixation can achieve and maintain satisfactory reduction, reducing the risk of axial reduction loss.Our clinical healing assessment criterion involved x-ray examination, which showed bridging callus formation in three directions at the fracture ends. We did not include cases of tibia fractures with severe open injuries classified as Gustilo-Anderson grade II and III in our study. There were no deep infections or osteomyelitis observed in our patients, possibly due to thorough debridement and appropriate antibiotic use. In the ESIN group, tibial shortening was observed due to factors such as axial displacement and angular deformities. At the final follow-up, LLD was found to be smaller in the E-K group compared to the ESIN group, but both groups had LLD less than 1 cm.The AOFAS functional evaluation score was 75.7% in the ESIN group and 100% in the E-K group, with a significant statistical difference (*P* = 0.027). This difference may be related to the noticeable coronal and sagittal angular deformities and poor alignment observed in the ESIN group. The E-K technique also faces some challenges: the addition of Kirschner wires may pose risks such as vascular and nerve damage, Kirschner wire displacement, needle tract infection, and the unknown potential for damage or interference with the distal tibial growth plate.

Our study has the following inherent limitations. The study is a clinical retrospective study with a relatively small sample size, which makes it difficult to avoid bias in the results. Further clinical data is needed to verify the results of this study. Additionally, this is a single-center study, and it is difficult to determine whether this has an impact on treatment outcomes. A multicenter study is needed to increase scientific validity and provide reference for the treatment of DTDMJ fractures in children.

## 5 Conclusion

E-K technique is a viable choice for the treatment of DTDMJ fractures in children, with proven clinical effectiveness. Its advantages include simple surgical procedures, safety, and reliability, with a low incidence of severe complications. However, further prospective research is needed to compare E-K with other treatment modalities.

## Data Availability

The original contributions presented in the study are included in the article/Supplementary Material, further inquiries can be directed to the corresponding author.
